# An acoustic platform for facile, size-targeted polymeric nanoparticle synthesis

**DOI:** 10.1039/d6sc03741k

**Published:** 2026-05-27

**Authors:** Keiran Mc Carogher, Lakshani J. Weerarathna, Tanja Junkers, Simon Kuhn

**Affiliations:** a Department of Chemical Engineering, KU Leuven Celestijnenlaan 200F 3001 Leuven Belgium simon.kuhn@kuleuven.be; b Polymer Reaction Design Group, School of Chemistry, Monash University Clayton VIC 3800 Australia tanja.junkers@monash.edu

## Abstract

A nanoparticle synthesis platform exploiting acoustic irradiation was developed and found to be capable of reproducibly synthesising particles of different sizes. In this platform, acoustic emulsification is used to generate dispersed droplets that are subsequently converted into solid nanoparticles upon solvent removal. This work shows that acoustic emulsification can be used to transform nanoparticle synthesis from a qualitative, empirically optimized technique into a predictive platform for size control by linking cavitation-driven droplet formation to simple, experimentally validated design rules. This size control was readily achieved through the variation of two parameters: acoustic frequency and polymer concentration. Changes in frequency produced substantial shifts in particle size, with lower frequencies (132 kHz) generating larger cavitation bubbles that collapse more violently and produce smaller dispersed droplets, while adjustments in polymer concentration allowed for finer tuning of particle size upon solvent removal. Across the investigated combinations of frequency and polymer concentration, particle sizes in the range of 51–177 nm were obtained. Under the investigated conditions, the estimated droplet size for a given frequency remained independent of polymer concentration, enabling the application of a simple mass balance to predict the polymer concentration required to obtain the desired particle size.

## Abstract 2

Acoustic frequency sets the droplet template, while polymer concentration tunes the final nanoparticle size, enabling predictable size-targeted synthesis.

## Graphical abstract

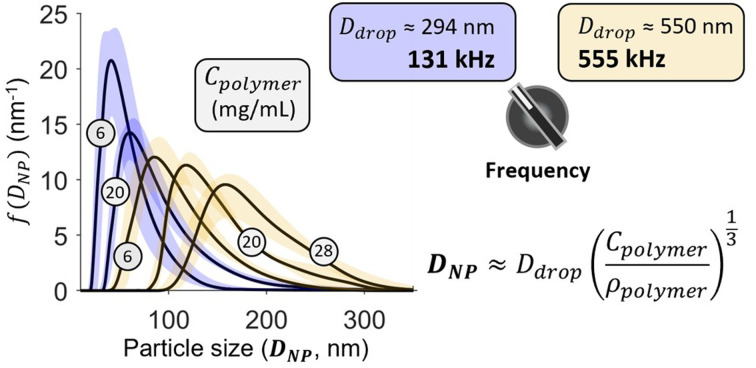

## Introduction

Nanoparticles have attracted significant attention across various fields due to their unique physicochemical properties that emerge at nanoscale.^1^ Their tuneable composition, size, and surface chemistry have enabled widespread use across sectors ranging from food and agriculture to advanced materials.^2–4^ In medicine in particular, these same attributes offer solutions to major limitations of conventional therapies by improving the pharmacokinetics and pharmacodynamics of drugs.^5–7^ More specifically, nanoparticle-based systems can enable sustained drug release, achieve targeted delivery, and protect therapeutic agents from premature degradation.^8^

Of the physicochemical properties, particle size is critical, as it strongly influences cellular uptake, particle toxicity, biodistribution, clearance and overall biological response.^1,5,9–12^

**Fig. 1:**
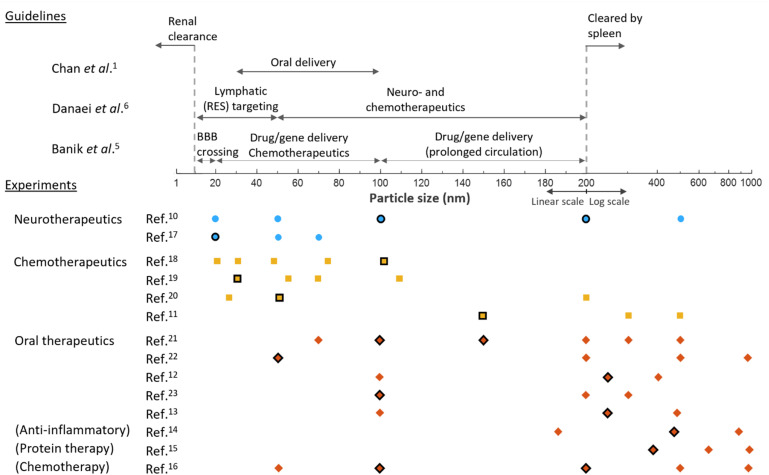
Top: Reported guidelines for effective nanoparticle size ranges across different delivery routes and therapeutic applications. Bottom: Representative experimental studies examining the effect of particle size in specific therapeutic contexts, with the size ranges identified by the authors as most effective highlighted in bold.

Consequently, several reviews have proposed guidelines outlining effective particle size ranges for different delivery routes and therapeutic applications.^1,5,6^Fig. 1 illustrates some of these guidelines, together with selected experimental studies that have examined the effect of particle size across diverse therapeutic contexts,^10–23^ primarily with the aim of using nanoparticles with specific functionalities for the intended application. The size ranges reported as most effective by the respective authors are highlighted in bold.

**Fig. 2:**
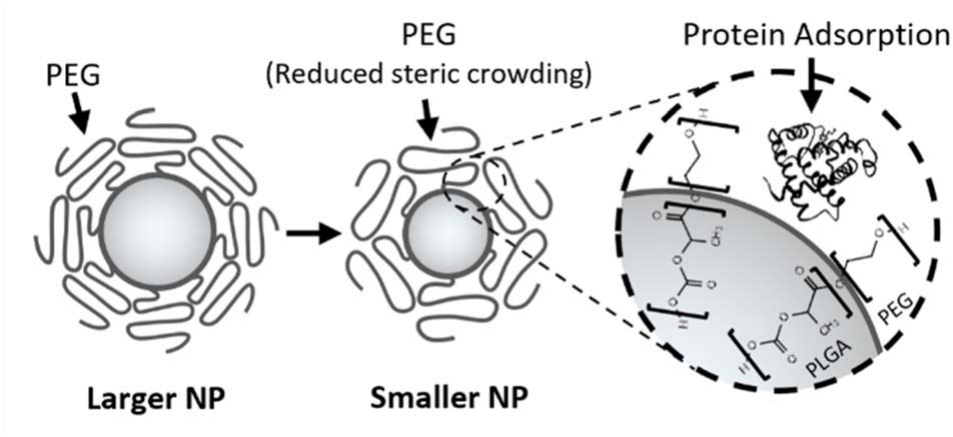
A schematic example of how particle size can influence nanoparticle interfacial behaviour. Reducing particle size increases surface curvature, which can alter the steric interactions of surface-bound functional groups such as PEG. This may reduce the thermodynamic barrier to protein adsorption, thereby compromising intended biological performance.

These studies illustrate that the effective nanoparticle size range is highly application dependent, since the physicochemical diversity that enables broad applications also contributes to the complexities of nano–bio interactions, which are not yet fully understood.^1,5^ In addition, particle size is not merely a geometric descriptor that influences transport and biodistribution, but also a design parameter that can alter both the particle's bulk^24^ and interfacial chemistry,^25^ with important consequences for biological fate and response, as illustrated in Fig. 2.^26^

Furthermore, to the best of our knowledge, systematic investigations across broad ranges of particle size, specifically designed with tailored functionalities, remain scarce. More commonly, researchers rely on commercially available particles of predefined size classes without functionalization,^10,16,21,22^ or on inorganic nanoparticles,^12,13,17,20^ to investigate size-dependent effects. As a result, progress in the field and a deeper understanding of the complex nano–bio interactions of functionalized polymeric nanoparticles are hindered without accessible platforms capable of reproducibly synthesizing particles of different sizes with defined functionalities,^15^ either in-house or at scale.

These challenges are rooted in the inherent complexity of nanoparticle formation, which is governed by both thermodynamic driving forces and kinetic constraints.^27^ While the underlying principles of nucleation and growth are well established, many existing synthesis platforms lack the spatial and temporal precision required to reproducibly synthesise particles with well-defined sizes and functionalities. For example, Streck *et al.*^28^ compared bulk nanoprecipitation with a microfluidic method for CPP-modified PLGA nanoparticles and found that, even after optimisation, bulk preparation still produced larger particles with higher batch-to-batch variability, whereas the microfluidic process yielded 150 nm nanoparticles with PDI < 0.15 and markedly improved reproducibility. Microfluidic flow platforms have therefore emerged as a promising approach, offering enhanced control at smaller length scales, enabling improved regulation of flow and rapid, well-defined mixing to establish more homogeneous conditions for particle formation.^29,30^

Despite these advantages, microfluidic technologies alone do not fully resolve these issues, as particle synthesis in conventional microfluidic systems remains a multivariate process in which many parameters are coupled, making it difficult to address specific particle characteristics independently and consistently across platforms.^31,32^ Moreover, characterising and modelling these processes is non-trivial, as the coupling between mixing phenomena, particle formation and growth remains difficult to capture quantitatively. A further limitation is that such well-controlled environments can typically only be maintained at very small production scales, and efforts to achieve higher productivities by either scaling up individual devices or operating multiple units in parallel often compromise control, leading to diminished reproducibility and poorly defined particle formation.

To circumvent these issues, a well-controlled environment is required for particle formation to occur. In such an environment, parameters that dictate specific particle characteristics, for example particle size, should ideally be decoupled, allowing these features to be tuned independently and with high reproducibility. Finally, to achieve higher throughput, this well-controlled environment must be readily replicable at scale. This would provide a more direct route to size targeting with improved consistency across platforms and at larger scales.

In this work, acoustic emulsification (AE) is explored as a means of establishing such a framework. By generating droplets through an externally applied acoustic field, AE offers a route to (predominantly) decouple particle size control from purely flow- and geometry-driven hydrodynamic effects. In this approach, an organic solvent containing dissolved polymer is acoustically dispersed into an immiscible aqueous phase within a microfluidic flow reactor, generating a population of droplets that subsequently serve as individual microenvironments for particle formation during solvent removal (SR). If each droplet yields a single solid particle, conservation of mass implies that the final particle size should be governed primarily by the droplet size and polymer concentration. Under these conditions, droplet formation and particle solidification can be conceptually separated, enabling more direct control over particle size and polydispersity.

This method, commonly referred to as the emulsion–solvent technique,^33^ has been widely used to produce polymeric nanoparticles with uniform sizes, low polydispersity, high drug loading, and sustained release behaviour.^14,23,34^ However, systematic studies of size control through acoustic emulsification remain limited, and the specific influence of acoustic parameters on droplet breakup and, consequently, final particle size is still not well understood. The present work aims to address this gap by examining the acoustic and flow parameters that govern droplet and, consequently, particle size, thereby establishing a platform for reproducible and facile synthesis of nanoparticles across a range of defined sizes.

**Fig. 3:**
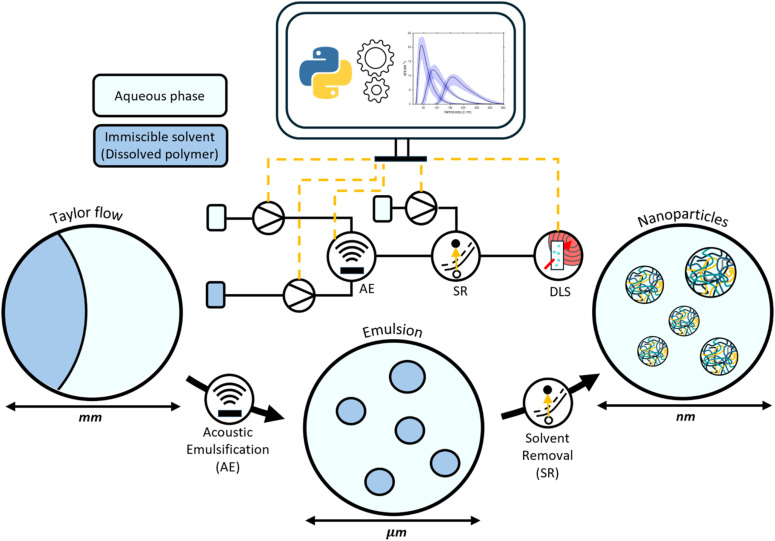
Schematic overview of the integrated experimental system used for size-targeted nanoparticle synthesis. Here, acoustic emulsification (AE) generates a dispersion of droplets containing dissolved polymer within a microfluidic reactor, which solidify into nanoparticles upon solvent removal (SR). The resulting particles are analysed using the automated high-throughput DLS platform to assess the influence of acoustic and flow parameters on particle size.

In doing so, this study provides an enabling tool to systematically synthesise and probe size-dependent interactions, to tailor nanoparticle formulations to specific therapeutic applications, and to inform the design of scalable processes that maintain precise size control at higher throughputs. To achieve this, the study leverages the automated high-throughput DLS-based screening platform developed by Weerarathna *et al.*,^31^ enabling variation of both acoustic and flow parameters for systematic exploration of the resulting parameter space. A schematic representation of the integrated experimental system is shown in Fig. 3.

## Experimental

**Fig. 4:**
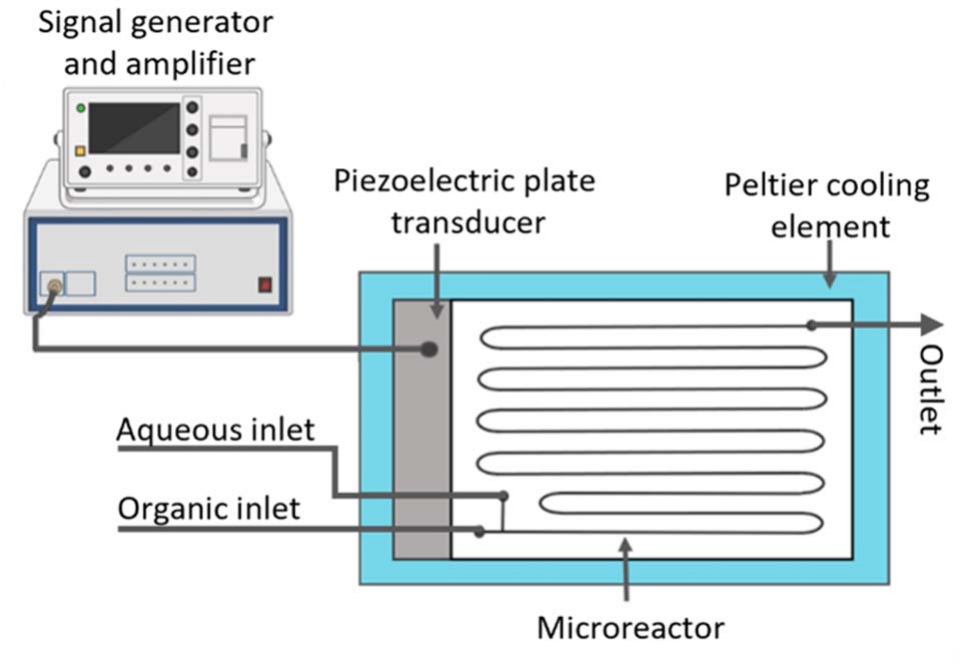
Schematic representation of the acoustic reactor used to generate the emulsions for nanoparticle formation.

A custom acoustic microreactor was used to generate the emulsion for nanoparticle formation. The reactor consisted of a water-jet cut glass microreactor (Little Things Factory GmbH, Germany, 1 mL, 2 × 1 mm^2^ square channels) coupled to a piezoelectric plate transducer (Pz26, Ferroperm; 80 × 40 × 4 mm^3^) which generated ultrasound within the microreactor channels. The reactor assembly was positioned on a Peltier cooling element (RS Components) connected to a DC power supply (Velleman), allowing active temperature control. The outlet temperature was monitored with a thermocouple and maintained at 30 °C during emulsification to maintain mild processing conditions relevant to hydrolytically degradable polymeric nanoparticle systems. A signal generator (33500B, Keysight) coupled with a power amplifier (AG 1006, T&C) was connected to the piezoelectric plate transducer to actuate ultrasound at the desired frequency and power. A schematic representation of the acoustic reactor used is shown in Fig. 4. Further details on the acoustic microreactor system can be found in ref. 35 and 36.

Fluid delivery was controlled using three peristaltic pumps (Vapourtec, SF-10). The organic phase (polystyrene or PLGA dissolved in ethyl acetate at defined concentrations) was fed to the first inlet of the acoustic reactor. The aqueous phase containing surfactant (sodium dodecyl sulfate (SDS), 5 mM, fixed concentration) was supplied to the second inlet of the acoustic reactor. The organic and aqueous phases were introduced directly into the reactor through separate inlets and were not premixed prior to acoustic treatment, such that a segmented two-phase flow was established.

Nanoparticles were generated through two consecutive processes. First, the organic phase was acoustically emulsified into the aqueous continuous phase inside the reactor. Second, the outlet emulsion was quenched at a downstream T-junction using an additional aqueous stream delivered by a third pump. Because ethyl acetate is partially miscible with water, this quenching step induced solvent displacement and consequent nanoparticle formation. The quench flow rate was adjusted to maintain an aqueous-to-organic volume ratio of 34 : 1, which was sufficient to ensure complete displacement of ethyl acetate based on its solubility in water (∼74 g dm^−3^).

The quenched outlet stream was fed to the DLS (Litesizer, Anton Paar) flow cell (50 µL, 1.5 mm pathlength, Hellma) for inline particle size measurements. All components were connected using PFA tubing (IDEX, ID 0.5 mm, OD 1/16″). The intensity-weighted mean hydrodynamic diameters (MHD) and polydispersity indices (PDI) were obtained using a standard cumulant model (ISO 22412), while the volume-weighted MHD values were determined using a non-negative least squares algorithm with Tikhonov regularisation. For all DLS measurements, the dispersing medium was set to water (refractive index 1.33) and the particle material to polystyrene (refractive index 1.59). The reported averages and standard deviations were obtained from at least three measurements.

A Python script written by Weismantel and Weerarathna *et al.*^31,32^ was used to control the flow and acoustic parameters, as well as the nanoparticle analysis (DLS) platform. In total, the influence of ultrasound frequency (*f* = 131, 330, 555 kHz), acoustic power (*P* = 5, 10, 15 W), inlet aqueous feed fraction (*β*_Aq_ = 0.70, 0.8, 0.9, 0.95), residence time (*τ* = 4, 8, 12 min), and polymer concentration (*C*_PS_ = 6, 12, 20, 28 mg mL^−1^) was systematically investigated using the automated platform.

The aqueous feed fraction *β*_Aq_ is calculated as1
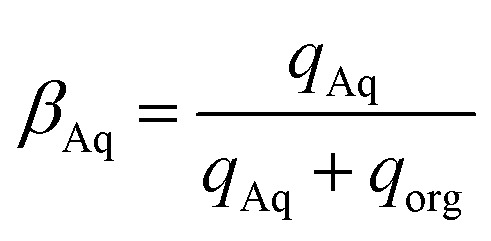
with *q*_Aq_ and *q*_org_ the aqueous and organic reactor inlet volumetric feed rates respectively. Residence time was controlled by varying the total volumetric flow rate (*q*_Aq_ + *q*_org_) through the reactor and estimated assuming plug-flow behaviour.

Under the acoustic conditions used here, significant polymer degradation is not expected. This is consistent with GPC analysis performed on the same acoustic reactor platform, which showed no significant change in PLGA molecular weight distribution after representative acoustic exposure.^35^

Polystyrene (PS, *M*_n_ = 48 000 g mol^−1^) was synthesized by free-radical polymerization of styrene (S, 99% Merck) using 1,1′-azobis(isobutyronitrile) (AIBN, 98%) as thermal initiator. Further details on the polystyrene synthesis and characterisation procedures are provided in the SI (Section S1). AIBN, surfactant sodium dodecyl sulfate (SDS, ≥ 99%), ethyl acetate (≥99.5%), hexane (≥99) and PLGA (Resomer® RG 504 H, acid terminated, L : G 50 : 50, *M*_n_ = 38 000–54 000 g mol^−1^) were purchased from Sigma-Aldrich.

## Results and discussion

Polystyrene was first used as a model polymer for systematic screening and mechanistic analysis, after which PLGA was used to assess whether the observed trends were transferable.

For each experimental condition, the automated platform was operated at defined organic and aqueous flow rates, with the organic phase containing polymer at the specified concentration. Within the acoustic reactor, the organic stream was emulsified into the aqueous surfactant phase, and the resulting droplets were converted downstream into nanoparticles by addition of a second aqueous quenching stream that induced solvent displacement. For each combination of acoustic conditions (frequency and power), flow conditions (residence time and aqueous feed fraction), and polymer concentration, the outlet nanoparticle suspension was analysed inline by DLS to obtain the mean hydrodynamic diameter and polydispersity, thereby directly linking processing conditions to particle size.

**Fig. 5:**
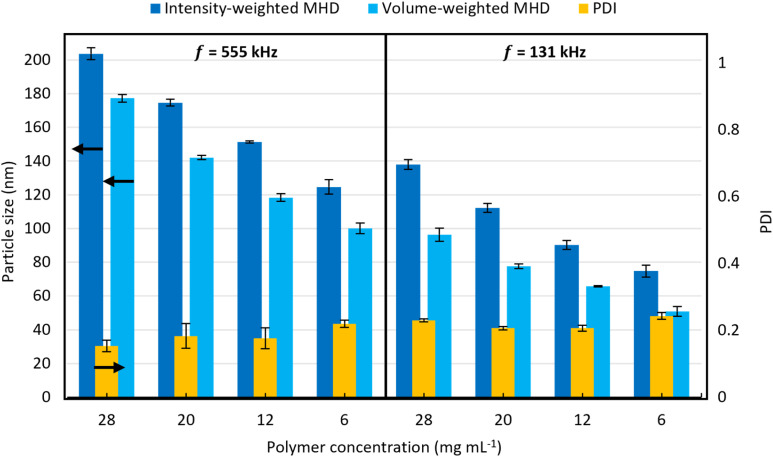
Effect of acoustic frequency (*f*) and polymer concentration (*C*_PS_) on polystyrene particle size for a reactor residence time (*τ*) of 12 minutes, aqueous feed fraction (*β*_Aq_) of 0.8 and an acoustic power (*P*) of 15 W. The error bars represent the standard deviation between three measurements. The corresponding intensity-weighted and volume-weighted DLS size distributions are shown in Fig. S4 in the SI (Section S2).

To test the central hypothesis that particle size is governed primarily by acoustic conditions, which determine droplet size, together with polymer concentration through conservation of mass, we first examined their combined effect on nanoparticle size. Consistent with this hypothesis, nanoparticle size was found to primarily depend on ultrasound frequency (*f*) and polymer concentration (*C*_PS_). Fig. 5 compares the resulting particle sizes obtained at 555 kHz and 131 kHz for polystyrene concentrations of 6, 12, 20, and 28 mg mL^−1^. The corresponding intensity- and volume-weighted DLS size distributions are provided in Fig. S4 of the SI (Section S2). At the same polymer concentration, operation at 131 kHz produced significantly smaller nanoparticles than at 555 kHz. While at a fixed frequency, the resulting particle size decreases with decreasing polymer concentration.

These observations indicate that multiple particle-size classes can be accessed readily by controlled variation of only these two parameters. The reduction in particle size with decreasing polymer concentration is attributed to the lower amount of polymer present in each droplet, which upon solidification yields smaller particles. Likewise, the reduction in particle size observed at lower frequencies is attributed to a decrease in droplet size, which for a given polymer concentration would also result in smaller particles due to the reduced polymer mass per droplet.

To assess whether the smaller particles obtained at lower frequency indeed arise from smaller droplets, droplet size distributions were first measured in the absence of dissolved polymer. Direct characterisation of the ethyl acetate–water system, however, proved difficult because ethyl acetate is partially miscible with water. Addition of the quenching stream led to partial dissolution of the dispersed phase, altering the apparent droplet-size distribution. Measurements on the unquenched outlet stream were also not possible, as the emulsion leaving the reactor was too concentrated and optically turbid for reliable DLS analysis.

To circumvent this limitation, hexane was used as an alternative dispersed phase because of its much lower solubility in water. Under otherwise comparable conditions, droplet sizes could then be measured after quenching in a hexane–water system. The results, summarised in Table 1, show that lower frequency again gave smaller droplets, supporting the interpretation that the frequency-dependent particle-size trends arise from differences in droplet breakup.

**Table 1:** Intensity-weighted droplet diameters (*D*^I^_D_) obtained for the hexane-water system at reactor residence time (*τ*) of 18 minutes, aqueous feed fraction (*β*_Aq_) of 0.95 and an acoustic power (*P*) of 15 W

*f* (kHz)	*D* ^I^_D _(nm)	PDI
131	582 ± 4	0.09 ± 0.06
555	754 ± 9	0.21 ± 0.03

Assuming conservation of polymer mass during solidification, and approximating each nanoparticle as a single solid sphere of uniform density originating from a single emulsified droplet, the corresponding droplet diameters were estimated from the mean volume-weighted MHDs in Fig. 5. Using a polymer density of 1.04 g cm^−3^, the estimated droplet sizes for each polymer concentration are summarised in Table 2. For both frequencies, the estimated droplet size remained approximately constant across the investigated concentration range, suggesting that single particles are formed from individual droplets and that droplet formation is largely independent of polymer concentration under the applied conditions.

**Table 2:** Droplet diameters (*D*_D_) estimated from the volume-weighted particle diameters (*D*^V^_P_) obtained for the ethyl acetate–water system (Fig. 5)

*C*_PS_ (mg mL^−1^)	*f* (kHz)			
	131		555	
	*D* ^V^_P_ (nm)	*D*_D_ (nm)	*D* ^V^_P_ (nm)	*D*_D_ (nm)
6	51	279	100	557
12	66	288	118	522
20	78	287	142	530
28	96	320	177	591
Mean *D*_D_ (nm)		294 ± 16		550 ± 27

Although the absolute droplet sizes measured in the hexane–water system differ from those estimated for the ethyl acetate–water system, the same qualitative trend was observed in both cases, namely the formation of smaller droplets at lower frequency. The discrepancy in absolute values can be attributed to the differences in interfacial tension and water miscibility between the two solvent systems, as well as to the different nature of the measurements, since the hexane droplets were characterized directly in a liquid–liquid emulsion, whereas the ethyl acetate-based droplet sizes were inferred indirectly from solid particles after solvent removal. A broader systematic evaluation of organic-phase selection, although clearly important for acoustic emulsification, lies beyond the scope of the present study.

Representative STEM images provided in the SI (Section S2) qualitatively confirm nanoparticle formation and are consistent with approximately spherical particle morphology, while showing no obvious evidence of hollow or highly porous structures.

Having identified ultrasound frequency (*f*) as the dominant parameter controlling particle size, the effects of acoustic power (*P*), aqueous feed fraction (*β*_Aq_), and residence time (*τ*) were further investigated with respect to frequency to determine whether similar trends were observed irrespective of it. This approach aimed to assess the extent to which these parameters are decoupled from frequency-dependent effects. The results are summarised in Tables 3–5, which present the effects of power (*P*), residence time (*τ*), and aqueous feed fraction (*β*_Aq_), respectively. For these experiments the polymer concentration was fixed at 12 mg mL^−1^.

The results indicate that, for a given frequency, both particle size and polydispersity tend to decrease with increasing acoustic power, longer residence time, and higher aqueous feed fractions, except at the highest feed fractions, where deviations arise due to the partial miscibility of ethyl acetate in water, which will be discussed shortly. However, the magnitude of these effects is considerably smaller than that observed when varying the ultrasound frequency.

At lower powers, shorter residence times, and/or lower aqueous feed fractions, emulsification was incomplete. This is confirmed visually by the presence of larger, unbroken organic slugs exiting the reactor prior to quenching, which would ultimately result in the formation of larger particles and broader size distributions.

These experimental trends can be understood by considering the underlying mechanisms of acoustic emulsification, which relies on the transfer of acoustic energy into the fluid to generate the hydrodynamic stresses required for deforming and rupturing immiscible interfaces. Of the mechanisms in which acoustic energy is transferred, for the generation of fine dispersed droplets in particular, breakup is largely considered to be driven by the violent oscillation and collapse of cavitation bubbles resulting in the formation of liquid jets and intense shockwaves which break apart the liquid–liquid interface.^37–39^

For a spherical droplet of radius *R*_D_, the pressure emitted from a cavitation bubble must at least exceed the Laplace pressure (*p*_L_) for droplet fragmentation to become possible:^40^2
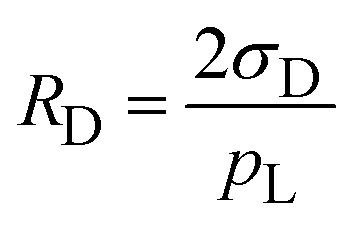
with *σ*_D_ the droplet-continuous phase interfacial tension. From this relationship it follows that higher emitted acoustic pressures promote the formation of smaller droplets. Given that the pressure emitted from a collapsing cavitation bubble is proportional to the maximum internal gas pressure (*p*_max_) reached at the moment of collapse,^37^ the relationship between ultrasound parameters such as frequency (*f*) and power (*P*) can be evaluated using the expression for *p*_max_ as presented by Leighton.^41^ The full derivations are provided in the SI (Section S3), from these relations, it follows that the maximum internal pressure, and consequently the emitted pressure, scales with frequency accordingly:3
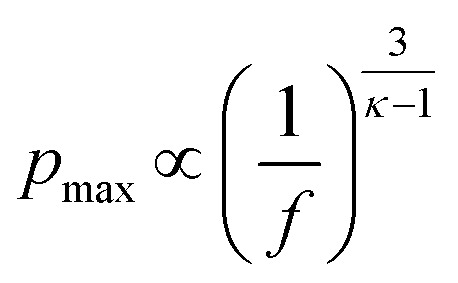


**Fig. 6:**
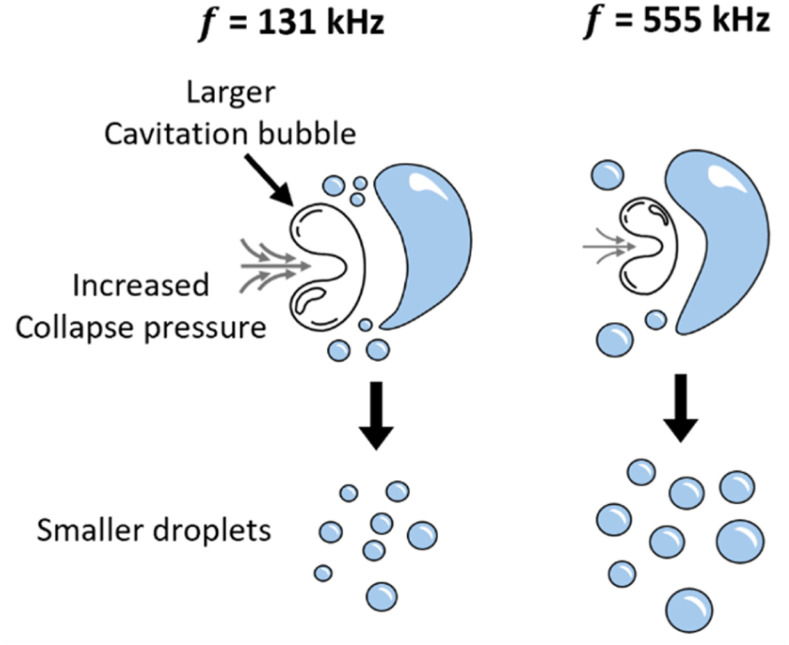
Schematic illustration of the proposed effect of ultrasound frequency on cavitation-bubble collapse and droplet breakup. At lower frequency (131 kHz), cavitation bubbles grow to larger sizes before collapsing more violently, generating higher pressure pulses and yielding smaller droplets. At higher frequency (555 kHz), cavitation-bubble collapse is less violent, producing lower pressure pulses and larger droplets.

This implies that, at lower frequencies, cavitation bubbles collapse more violently and emit stronger pressure pulses, leading to more intense emulsification and consequently smaller droplet sizes, as schematically illustrated in Fig. 6, a trend corroborated by experimental observations.^42,43^

With respect to the influence of the acoustic pressure amplitude (*p*_A_) on the maximum collapse pressure (*p*_max_), eqn (S1)–(S8) show that for high acoustic pressures (*p*_A_), *p*_max_ scales with *p*_A_ accordingly:4
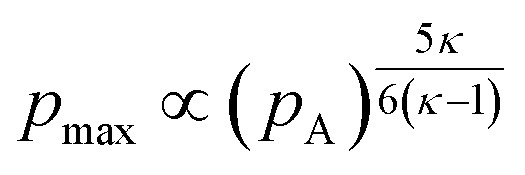


These relationships indicate that, although the maximum collapse pressure (*p*_max_) increases with acoustic pressure amplitude (*p*_A_), the influence of frequency (*f*) on *p*_max_ is stronger for realistic polytropic indices (*κ* < 3.6), particularly for air bubbles, where *κ* typically lies between 1.0 and 1.4.^41^ This explains why frequency exerts a more pronounced effect on the minimum attainable droplet size than comparable changes in power.

Furthermore, in practice, increasing power is also subject to diminishing returns. Acoustic energy is dissipated through viscous losses in the surrounding liquid and thermal losses within collapsing cavitation bubbles, so increasing the input power does not necessarily translate directly into a proportional increase in effective acoustic drive. This self-saturation behaviour is well documented in cavitating systems.^44,45^ Together with practical limitations such as cooling requirements and transducer operating limits, this makes frequency the more effective variable for particle-size control.

Nevertheless, sufficient acoustic power delivery remains essential to generate the acoustic pressures required for cavitation collapse and to sustain high populations of active cavitation bubbles, both of which are crucial for effective size control *via* acoustic frequency. Acoustic power directly influences the emulsification rate of the dispersed phase. As shown in Table 3, at lower acoustic powers (*P*) the residence time (*τ*) is insufficient to achieve complete emulsification, leading to larger particles and higher polydispersity.

**Table 3:** Effect of acoustic power (*P*) and on particle size (*D*^I^_P_) for a reactor residence time (*τ*) of 4 minutes and aqueous feed fraction (*β*_Aq_) of 0.8

*P* (W)	*f* (kHz)					
	131		330		555	
	*D* ^I^_P_ (nm)	PDI	*D* ^I^_P_ (nm)	PDI	*D* ^I^_P_ (nm)	PDI
5	—	—	201 ± 13	0.23 ± 0.02	219 ± 28	0.24 ± 0.01
10	71 ± 16	0.26 ± 0.01	180 ± 16	0.21 ± 0.03	208 ± 7	0.24 ± 0.01
15	51 ± 12	0.26 ± 0.01	171 ± 11	0.2 ± 0.02	198 ± 9	0.2 ± 0.03

Similarly, for a given acoustic power (*P*) and frequency (*f*), as well as dispersed-phase feed fraction (1 − *β*_Aq_), sufficient residence time (*τ*) is also required to achieve complete emulsification, reaching what is likely the minimum threshold droplet size, as shown in Table 4.

**Table 4:** Effect of reactor residence time (*τ*) on particle size (*D*^I^_P_) for an aqueous feed fraction (*β*_Aq_) of 0.8 and an acoustic power (*P*) of 15 W

*τ* (min)	*f* (kHz)					
	131		330		555	
	*D* ^I^_P_ (nm)	PDI	*D* ^I^_P_ (nm)	PDI	*D* ^I^_P_ (nm)	PDI
4	101	0.25	158	0.25	198	0.2
8	87	0.2	156	0.26	178	0.22
12	90	0.21	178	0.23	151	0.15

Increasing the aqueous feed fraction (*β*_Aq_) resulted in smaller particle sizes and lower PDI values for ratios below 0.9, Table 5. This trend is also linked to the emulsification rate, as higher dispersed-phase feed fractions (1 − *β*_Aq_) require longer residence times (*τ*) to reach complete emulsification. However, at high aqueous feed fractions (*β*_Aq_ ≥ 0.9), both particle size and polydispersity increased. This increase is attributed to the partial miscibility of ethyl acetate in water. At such high ratios, a significant fraction of the ethyl acetate dissolves within the acoustic reactor, an estimated 75% for aqueous to organic ratio of 0.9 (assuming a solubility of ∼74 g dm^−3^), causing premature nanoprecipitation of the non-emulsified organic phase and yielding larger, more polydisperse particles.

**Table 5:** Effect of aqueous feed fraction (*β*_Aq_) on particle size (*D*^I^_P_) for reactor residence time (*τ*) of 4 minutes and an acoustic power (*P*) of 15 W

*β*_Aq_	*f* (kHz)					
	131		330		555	
	*D* ^I^_P_ (nm)	PDI	*D* ^*I*^_P_ (nm)	PDI	*D* ^*I*^_P_ (nm)	PDI
0.7	132	0.22	144	0.23	181	0.14
0.8	75	0.22	171	0.2	165	0.19
0.9	250	0.29	467	0.24	172	0.24
0.95	296	0.25	520	0.285	278	0.18

Taken together, these results suggest a practical operating strategy for the platform. First, the aqueous feed fraction should be chosen such that solvent dissolution within the acoustic reactor remains limited, thereby avoiding premature nanoprecipitation. Once this condition is satisfied, ultrasound frequency can be used to select the principal particle-size regime, while polymer concentration provides finer control within that regime. Acoustic power should then be set sufficiently high to promote efficient emulsification, while remaining within the practical operating limits of the system, since it primarily influences the rate of emulsification and therefore process productivity. Finally, the residence time should be sufficient to ensure complete emulsification under the chosen conditions, so that particle size remains governed by frequency and polymer concentration rather than by incomplete droplet breakup.

Finally, to verify whether the frequency-dependent size trends were transferable across polymer systems, PLGA was examined in place of polystyrene at a concentration of 12 mg mL^−1^. The resulting particle sizes obtained at 133 and 555 kHz are shown in Table 6. The results confirm that effective size control through acoustic frequency can be achieved for both polymers, demonstrating that the principal size-control trend is not limited to polystyrene and is retained for a pharmaceutically relevant biodegradable polymer.

**Table 6:** Effect of acoustic frequency (*f*) on PLGA particle size (*D*^I^_P_) for reactor residence time (*τ*) of 18 minutes, aqueous feed fraction (*β*_Aq_) of 0.8 and an acoustic power (*P*) of 15 W

*f* (kHz)	*D* ^I^_P_ (nm)	PDI
133	88 ± 2	0.25 ± 0.04
555	148 ± 5	0.19 ± 0.03

## Conclusions

The results demonstrate that the proposed acoustic platform provides a well-controlled and replicable environment for polymer nanoparticle formation, in which the principal size-determining parameters are largely decoupled.

In this platform, acoustically generated droplets act as independent microenvironments for particle formation, such that final particle size can be targeted through control of droplet size and polymer concentration, rather than through tight coupling of hydrodynamics, particle nucleation, and growth.

Within the parameter space examined here, ultrasound frequency was the dominant variable governing the minimum attainable droplet size, provided that sufficient residence time and acoustic power are supplied to achieve complete emulsification. This observation is consistent with the cavitation-based theoretical analysis presented by Leighton,^41^ which predicts more violent bubble collapse and higher emitted pressures at lower frequencies, thereby enabling the formation of smaller droplets and, consequently, smaller particles.

Consequently, two of the investigated frequencies produced particles in two distinct and well-separated size ranges. Intermediate particle sizes between these ranges could then be achieved by adjusting the polymer concentration. Under the conditions examined in this work, the estimated droplet size appeared largely independent of polymer concentration. Therefore, for a given frequency, and thus a given droplet size, a simple mass balance can be applied to estimate the polymer concentration required to obtain the desired particle size.

Together, these findings demonstrate that the developed acoustic platform, along with the insights gained, provides an accessible and reproducible means of synthesizing particles across a range of sizes, enabling deeper investigations into the complex nano–bio interactions of functionalized polymeric nanoparticles.

More broadly, the present acoustic emulsification strategy may be extendable to other nanoparticle systems beyond the polymeric particles studied here, provided that the phase configuration and formation mechanism are adapted accordingly. The broader utility of the platform therefore lies in its use as a controllable droplet-generation step that may be coupled to different downstream solidification strategies, including those better suited to achieving higher final product concentrations, or to self-assembly routes.

## Author contributions

K. M.: conceptualization, methodology, investigation, formal analysis, data curation, visualization, writing – original draft, review & editing. L. W.: software, data curation, writing – review & editing. T. J.: supervision, administration, resources, writing – review & editing. S. K.: conceptualization, administration, supervision, resources, funding acquisition, writing – review & editing.

## Conflicts of interest

There are no conflicts to declare.

## Supplementary Material

SC-017-D6SC03741K-s001

## Data Availability

The data supporting the findings of this study are available within the article and its supplementary information (SI). Additional raw data are available from the corresponding author upon reasonable request. Supplementary information is available. See DOI: https://doi.org/10.1039/d6sc03741k.
