# Supplementary material for: An acoustic platform for facile, size-targeted polymeric nanoparticle synthesis
Source: Chem Sci. 2026 May 27;17(26):13139–47. doi: 10.1039/d6sc03741k (PMC13215118; doi:10.1039/d6sc03741k)
Supplement: SC-017-D6SC03741K-s001 [file SC-017-D6SC03741K-s001.pdf]

Supplementary Information (SI) for Chemical Science.
This journal is © The Royal Society of Chemistry 2026

# Electronic Supplementary Information

## An Acoustic Platform for Facile, Size-Targeted Polymeric Nanoparticle Synthesis

Keiran Mc Carogher[a], Lakshani J. Weerarathna[b], Tanja Junkers[b] and Simon Kuhn[a]
[a] Department of Chemical Engineering, KU Leuven, Celestijnenlaan 200F, 3001 Leuven, Belgium
[b] Polymer Reaction Design Group, School of Chemistry, Monash University, Clayton, VIC 3800, Australia

**Table of content**

- S1 Polystyrene synthesis and characterization - SEC profile and $^1$H NMR spectrum (Figure S1, S2) S-2
- S2 Particle characterization - STEM images, intensity-weighted and volume-weighted size distributions (Figure S3, S4) S-2
- S3 Dependence of maximum collapse pressure on ultrasound frequency and acoustic pressure amplitude – Leighton's analysis S-4

**S1 Polystyrene synthesis and characterisation**

In the synthesis of polystyrene, the thermal initiator 1,1′-azobis(isobutyronitrile) (AIBN, 98%, Sigma-Aldrich) was recrystallized in methanol prior to use. The monomer styrene (S, 99.9%, Sigma-Aldrich) was deinhibited over a column of activated basic alumina prior to use. The solvents toluene (Merck), N,N-dimethylformamide (DMF, Ajax Finechem), methanol (Ajax Finechem), and chloroform-d ($CDCl_3$, Deutero GmbH) were used as received.

Free-radical polymerization of styrene (S) was performed using classical batch-mode synthesis. In a 100 mL round-bottom flask, 192 mmol (20 g, 1 equiv., 4 M) of styrene and 0.19 mmol (0.03 g, 0.001 equiv.) of AIBN were dissolved in 25 mL of toluene. The flask was then purged with argon for 30 min. The solution was allowed to react at 75 °C in an oil bath under magnetic stirring for 24 h. The reaction was quenched by lowering the temperature and exposing the reaction mixture to air. The synthesized polymer was purified by precipitation into cold methanol. The final product was dried in vacuo for 24–48 h.

Molar mass distributions were analysed using a PSS SECcurity² GPC system operated with PSS WinGPC software, equipped with an autosampler, an SDV 5.0 µm guard column (50 × 8 mm), followed by three SDV analytical 5.0 µm columns with varying porosity (1000 Å, 100000 Å and 1000000 Å) (300 × 8 mm), coupled to a differential refractive index (RI) detector and viscosity detector, using tetrahydrofuran (THF) as the eluent at 40 °C with a flow rate of 1 mL $min^{-1}$. Toluene was used as the flow marker. The GPC system was calibrated using linear narrow polystyrene standards from PSS Laboratories ranging from 682 to $2.52 \times 10^6$ g $mol^{-1}$ PS ($K = 14.1 \times 10^{-5}$ dL $g^{-1}$ and $\alpha = 0.70$, THF, 30 °C). Molar masses of the samples were determined by universal calibration using Mark–Houwink–Kuhn–Sakurada (MHKS) parameters. The characterization data from SEC are shown in Figure S1.

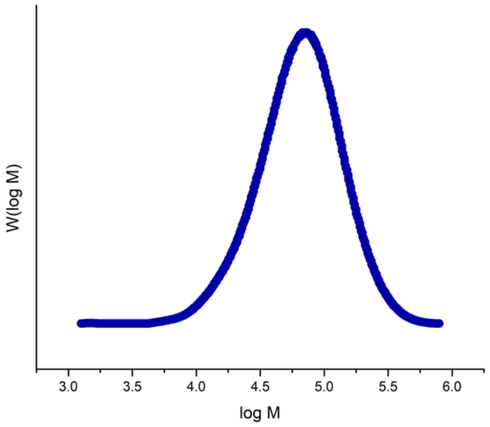


Figure S1. SEC profile of PS with $M_n$ = 48 000 g $mol^{-1}$ and dispersity (Đ) of 1.7.

Polystyrene samples were dissolved in $CDCl_3$, and $^1H$ NMR spectra were recorded on a Bruker Avance III 400 MHz NMR spectrometer and analysed using MestReNova software. The spectrum is shown in Figure. S2.

**S2 Particle characterisation**

All Scanning Transmission Electron Microscopy (STEM) images were taken on the Thermo Fisher Scientific Verios 5 UC field emission gun scanning electron microscope (FEG-SEM). It is an ultrahigh resolution FEG-SEM, including excellent low voltage performance, equipped with an Elstar electron column with UC+ technology. This system is fitted with a specimen quick loader and a multi-sample holding station, and for STEM, a special retractable STEM 3+ detector is used within the system.

The STEM samples were prepared on continuous carbon 200 mesh copper grids. A volume of 8 µL of the nanoaggregate solution was drop casted on to the grid. After 1 minute, it was blotted with filter paper without touching the grid to remove excess solvent. Then the staining of nanoparticles was done using a 1% w/w aqueous solution of uranyl acetate, where 3 µL of the staining agent was drop casted onto the sample loaded grid and after 20 seconds, the excess stain was carefully removed by blotting using a filter paper. It was dried in the fume hood overnight ensuring that the solvent was removed from the particles.

STEM images of the particles formed at 131 and 555 kHz for polystyrene (PS) concentrations of 6 and 28 mg $mL^{-1}$ are shown in Figure S3; for a reactor residence time of 12 minutes, aqueous feed fraction of 0.8 and an acoustic power of 15W. Images were taken in bright field (BF) and Dark field (DF) modes of detection at an accelerating voltage of 25.00 kV and a current of 6.3 pA.

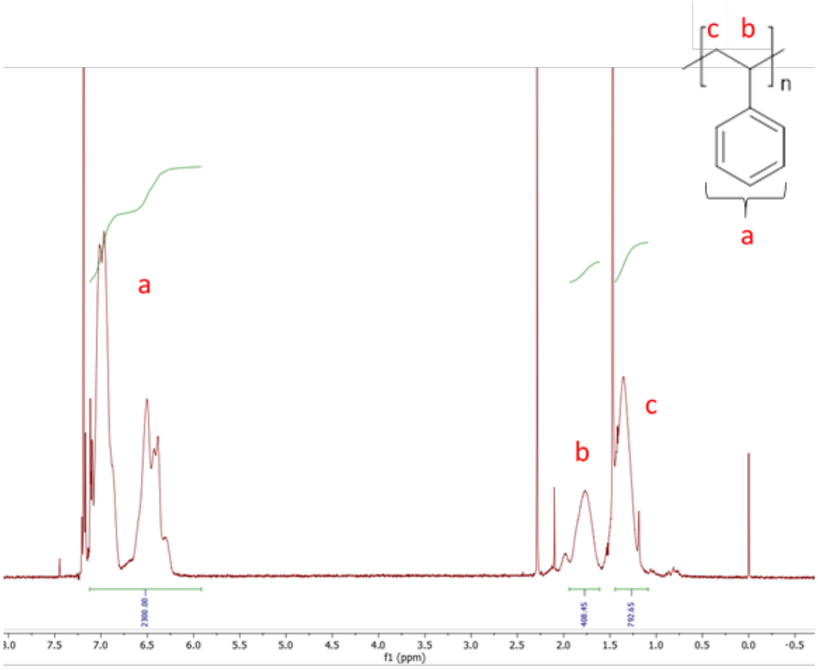


Figure S2. $^{1}H$ NMR spectrum of polystyrene (400 MHz, $CDCl_3$).

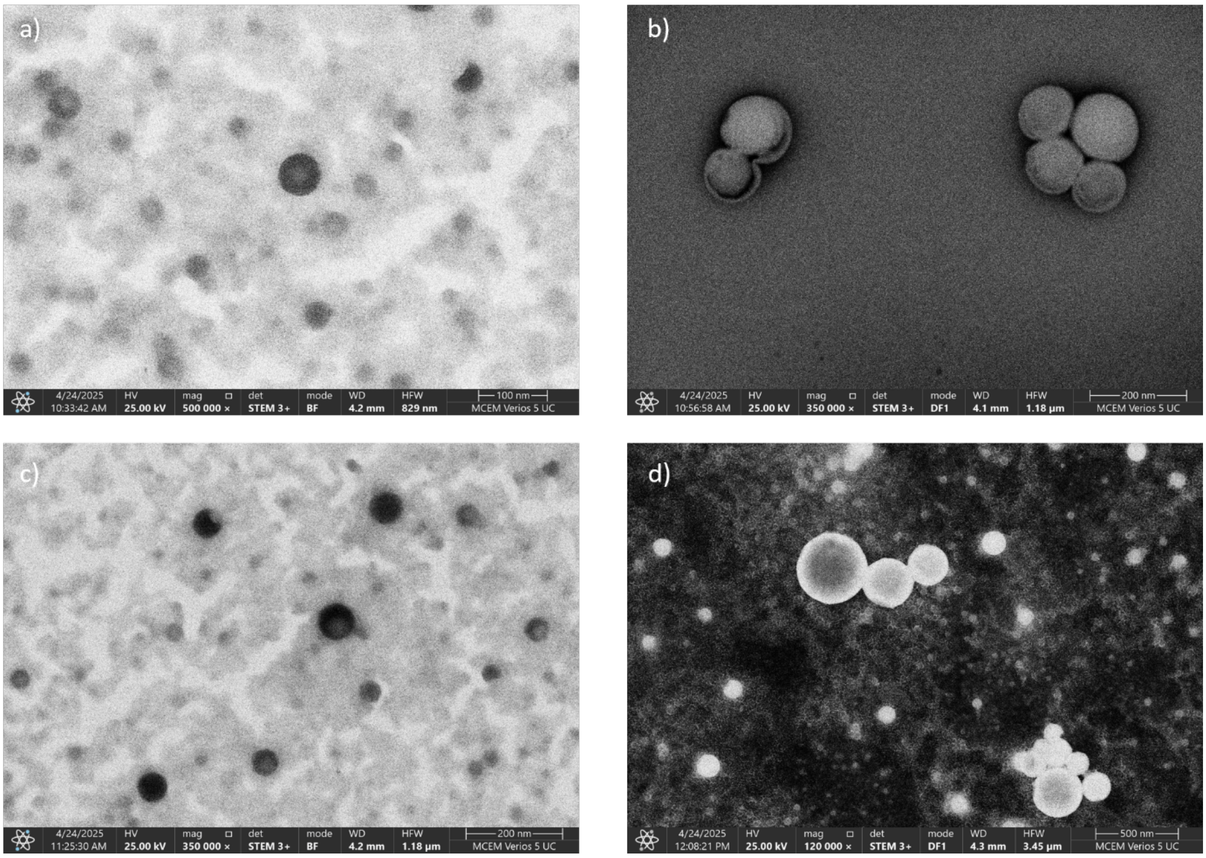


Figure S3: STEM images of polystyrene (PS) nanoparticles obtained at: a) 131 kHz and a PS concentration of 6 mg $mL^{-1}$ (BF), b) 131 kHz and 28 mg $mL^{-1}$ (DF), c) 555 kHz and 6 mg $mL^{-1}$ (BF), and d) 555 kHz and 28 mg $mL^{-1}$ (DF).

Figure S4 presents the dynamic light scattering (DLS) size distributions corresponding to the mean particle sizes shown in Figure 5 of the main text. Intensity-weighted and volume-weighted distributions are included for polystyrene nanoparticles prepared at 131 and 555 kHz for polymer concentrations of 6, 12, 20, and 28 mg mL$^{-1}$. All distributions were obtained using the Anton Paar analysis model based on a non-negative least squares algorithm with Tikhonov regularisation. The plotted curves represent the mean of three measurements, while the shaded regions denote the corresponding standard deviations. These distributions provide further detail on the size populations obtained under each condition and support the frequency- and concentration-dependent trends discussed in the main text.

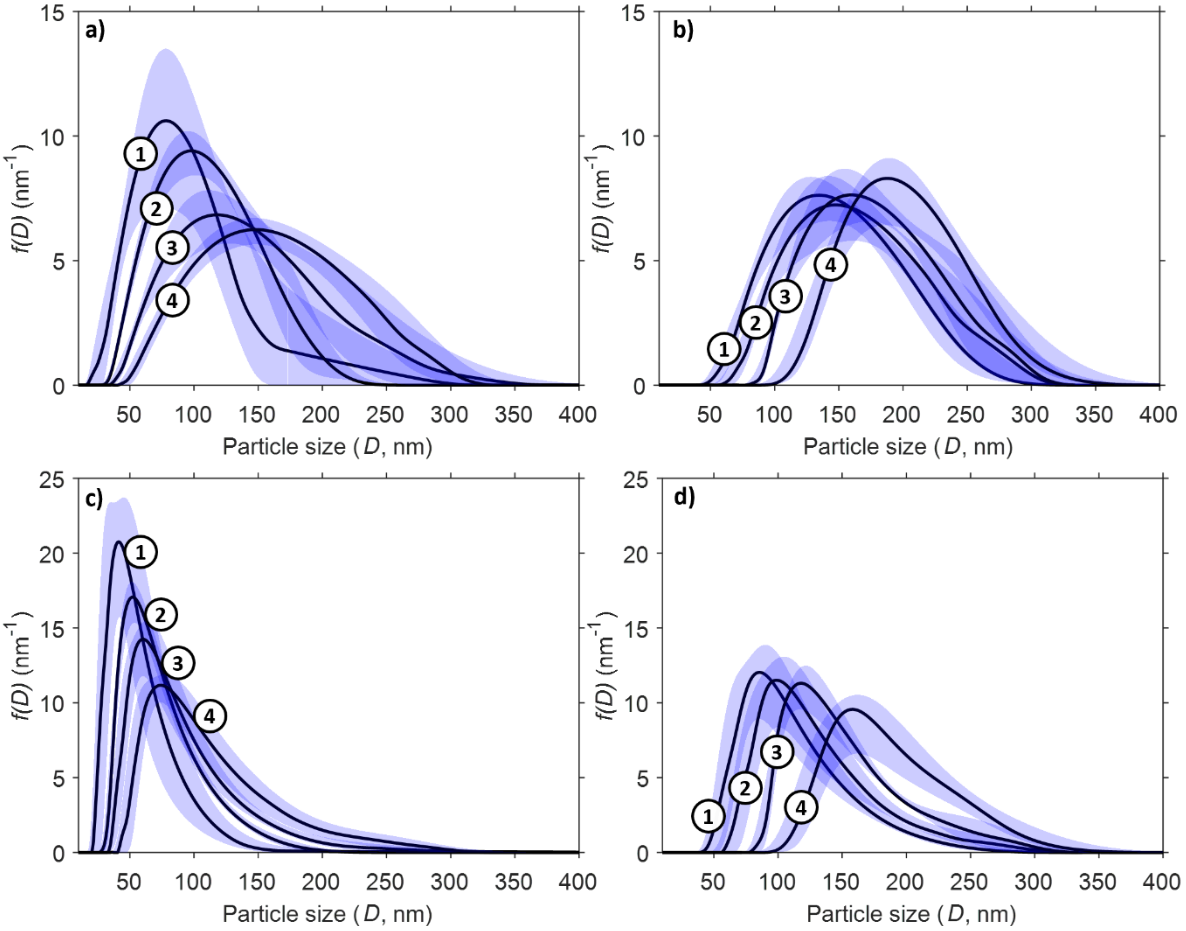


Figure S4: Dynamic light scattering (DLS) size distributions for polystyrene nanoparticles prepared at 131 and 555 kHz. (a) Intensity-weighted distributions at 131 kHz; (b) intensity-weighted distributions at 555 kHz; (c) volume-weighted distributions at 131 kHz; and (d) volume-weighted distributions at 555 kHz. All distributions were generated using the Anton Paar analysis model based on a non-negative least squares algorithm with Tikhonov regularisation. The plotted curves represent the mean of three measurements, while the shaded blue regions denote the corresponding standard deviation. Labels 1–4 correspond to polymer concentrations of 6, 12, 20, and 28 mg mL$^{-1}$, respectively.

**S3 Dependence of maximum collapse pressure on ultrasound frequency and acoustic pressure amplitude**

For a cavitation bubble initially in equilibrium with radius $R_0$, assuming the bubble expands slowly and isothermally from $R_0$ to a maximum radius prior to collapse ($R_{max}$ ), $p_{max}$ is given by:[1]

$$p_{max} = \left(\frac{R_{max}}{R_0}\right)^{\frac{3}{\kappa-1}} \left(p_0 \frac{2\sigma_c}{R_0}\right)^{-\frac{1}{\kappa-1}} (\kappa-1)^{\frac{\kappa}{\kappa-1}} (p_A + p_0)^{\frac{\kappa}{\kappa-1}} \tag{S1}$$

with $p_0$ the hydrostatic pressure, $\sigma_c$ the gas-liquid surface tension at the bubble interface, $p_A$ the acoustic driving pressure at the end of the compression cycle and $\kappa$ the polytropic index.

For cavitation bubbles with initial bubble radii ($R_0$) much smaller than the bubble resonance radius ($R_R$),

$$R_R = \frac{1}{2\pi f}\sqrt{\frac{3\kappa p_0}{\rho}} \tag{S2}$$

with $\rho$ the liquid density, the maximum radius prior to collapse ($R_0 \ll R_R$) can be approximated using:

$$R_{max} = \frac{2}{3\pi f}(p_A - p_0)\sqrt{\frac{2}{\rho\, p_A}}\left(1 + \frac{2}{3p_0}(p_a - p_0)\right)^{1/3} \tag{S3}$$

And for larger initial cavitation bubbles:

$$R_{max} = \left(R_0 + \sqrt{\frac{2\,\Delta p_{wall}}{3\rho}}\,t_{grow}\right)\left(1 + \frac{\Delta p_{wall}}{p_0}\right)^{1/3} \tag{S4}$$

where

$$t_{grow} = (t_1 - t_2) - (\Delta t_\sigma + \Delta t_I + \Delta t_\eta) \tag{S5}$$

$$t_1 \approx \frac{1}{2\pi f}\left(\frac{\pi}{2} - \sqrt{2\left(1 - \frac{p_B}{p_A}\right)}\right) \tag{S6}$$

$$t_2 \approx \frac{1}{2\pi f}\left(\frac{\pi}{2} - \sqrt{2\left(1 - \frac{p_0}{p_A}\right)}\right) \tag{S7}$$

$$\Delta p_{wall} \approx \frac{1}{3}\left(p_a + p_B - 2p_0 + \sqrt{(p_A - p_0)(p_A - p_B)}\right) \tag{S8}$$

with $\Delta t_\sigma$, $\Delta t_I$, $\Delta t_\eta$ the time delays caused by surface tension, inertia and viscosity respectively, and $p_B$ the Blake pressure threshold.

From these relations, it follows that for small initial bubbles $R_0$ << $R_R$ or $1/f$ >> $\frac{2\pi\left(R_0 - \sqrt{\frac{2\Delta p_{wall}}{3\rho}}(\Delta t_\sigma + \Delta t_I + \Delta t_\eta)\right)}{\sqrt{\frac{2\Delta p_{wall}}{3\rho}}\left(\sqrt{2(1-p_0/p_A)} + \sqrt{2(1-p_B/p_A)}\right)}$ the maximum internal pressure, and consequently the emitted pressure, scales with frequency accordingly:

$$p_{max} \propto \left(\frac{1}{f}\right)^{\frac{3}{\kappa-1}} \tag{S9}$$

With respect to the influence of the acoustic pressure amplitude ($p_A$) on the maximum collapse pressure ($p_{max}$), equations S1-8 show that for high acoustic pressures ($p_A$), $p_{max}$ scales with $p_A$ accordingly:

$$p_{max} \propto (p_A)^{\frac{5\kappa}{6(\kappa-1)}} \tag{S10}$$

for $P_A$>>$3/2p_0$ and R0 << RR or $p_A$ >> $p_B$, $\left(\frac{3R_0\sqrt{\rho}}{2t_{grow}}\right)^2$

**References**

1 T. Leighton, *The Acoustic Bubble*, Elsevier, 1994.
